# Neuromuscular Electrical Stimulation: A New Therapeutic Option for Chronic Diseases Based on Contraction-Induced Myokine Secretion

**DOI:** 10.3389/fphys.2019.01463

**Published:** 2019-11-28

**Authors:** Fabian Sanchis-Gomar, Sergio Lopez-Lopez, Carlos Romero-Morales, Nicola Maffulli, Giuseppe Lippi, Helios Pareja-Galeano

**Affiliations:** ^1^Department of Physiology, Faculty of Medicine, INCLIVA Biomedical Research Institute, University of Valencia, Valencia, Spain; ^2^Facultad de Ciencias del Deporte, Universidad Europea de Madrid, Madrid, Spain; ^3^Department of Musculoskeletal Disorders, Faculty of Medicine and Surgery, University of Salerno, Baronissi, Italy; ^4^Centre for Sports and Exercise Medicine, Barts and the London School of Medicine and Dentistry, Queen Mary University of London, London, United Kingdom; ^5^Guy Hilton Research Centre, School of Pharmacy and Bioengineering, Keele University, Stoke-on-Trent, United Kingdom; ^6^Section of Clinical Biochemistry, University of Verona, Verona, Italy

**Keywords:** electrotherapy, cytokines, transcutaneous electrical nerve stimulation, physical exercise, skeletal muscle contraction

## Abstract

Myokines are peptides known to modulate brain neuroplasticity, adipocyte metabolism, bone mineralization, endothelium repair and cell growth arrest in colon and breast cancer, among other processes. Repeated skeletal muscle contraction induces the production and secretion of myokines, which have a wide range of functions in different tissues and organs. This new role of skeletal muscle as a secretory organ means skeletal muscle contraction could be a key player in the prevention and/or management of chronic disease. However, some individuals are not capable of optimal physical exercise in terms of adequate duration, intensity or muscles involved, and therefore they may be virtually deprived of at least some of the physiological benefits induced by exercise. Neuromuscular electrical stimulation (NMES) is emerging as an effective physical exercise substitute for myokine induction. NMES is safe and efficient and has been shown to improve muscle strength, functional capacity, and quality of life. This alternative exercise modality elicits hypertrophy and neuromuscular adaptations of skeletal muscles. NMES stimulates circulating myokine secretion, promoting a cascade of endocrine, paracrine, and autocrine effects. We review the current evidence supporting NMES as an effective physical exercise substitute for inducing myokine production and its potential applications in health and disease.

## Introduction

Functions classically attributed to skeletal muscle are movement and maintenance of posture, protection of vital organs, stimulation of blood and lymphatic circulation, and activation of metabolic pathways as the consequence of the large amount of energy consumed. More recently, this perspective has broadened, as contracting skeletal muscles have been shown to release molecules responsible for signal transmission to other tissues (Hawley et al., 2014). These molecules were referred to as “the work stimulus,” “the work factor” or “the exercise factor” (Pedersen et al., 2003). It was originally hypothesized that this exercise factor could be potassium ions, lactic acid, adenosine, interleukin (IL)-6 or other metabolites, until Pedersen et al. (2003) suggested in 2003 that cytokines – produced and released by skeletal muscles contraction and exerting their effects on other organs – should be named “myokines.”

Myokines are recognized as potential candidates to manage metabolic diseases through their capacity to modulate fuel oxidation, hypertrophy, angiogenesis, inflammatory processes, and extracellular matrix regulation (Leal et al., 2018; Piccirillo, 2019). Myokines may also influence the onset and the course of other diseases through their endocrine functions, as they interplay with body weight regulation, inflammation, insulin sensitivity, tumor growth, and cognitive function (So et al., 2014; Carson, 2017; Hoffmann and Weigert, 2017). Thus, myokines may exert beneficial effects on metabolic syndrome-related disorders such as obesity, insulin resistance and type-2 diabetes, dyslipidemia; cardiovascular diseases such as hypertension and coronary heart disease; breast and colon cancer; and neuropsychiatric diseases such as Alzheimer’s, Parkinson’s and depression (Sanchis-Gomar and Perez-Quilis, 2014; Sanchis-Gomar et al., 2014; Pareja-Galeano et al., 2015).

Myostatin was the first myokine identified in 2008 (Allen et al., 2008), and IL-6 was the first myokine found to be secreted into the bloodstream in response to muscle contraction (Pedersen and Febbraio, 2008). Since then, several hundred myokines have been described, including cytokines, small proteins (∼ 5–20 kDa) and proteoglycan peptides produced and released by contracting muscle cells via secretion of proteins that signal between muscle and the rest of the body (Pedersen et al., 2007; Catoire et al., 2014). Thus, these peptidic molecules are expressed, produced, and released by muscle fibers which thus exert autocrine, paracrine, and/or endocrine effects (Pedersen et al., 2003). The autocrine, paracrine and/or endocrine (systemic) actions of myokines occur at picomolar concentrations (Pedersen and Febbraio, 2012; Pedersen, 2013). The autocrine and paracrine effects of myokines are mainly involved in the regulation of muscle physiology, muscle growth or lipid metabolism. However, myokine receptors have been identified in different tissues and organs, including the muscle itself, adipose tissue, liver, pancreas, bone, brain, heart, vessels, and immune cells, thereby modulating a myriad of functions (Pedersen et al., 2007; Lee and Jun, 2019).

Plasma levels of most myokines depend on the amount of contracted muscle mass and are hence strongly related to the amount of muscle mass exercised (Pedersen and Febbraio, 2008; Hody et al., 2019). For instance, IL-6 production is sensitive to exercise intensity (Ostrowski et al., 2000), an indirect measure of the muscle mass involved in contractile activity (Pedersen and Febbraio, 2008). Contracting skeletal muscle is an important source of plasma IL-6 (Steensberg et al., 2000; Fischer et al., 2004), and exercise involving a limited muscle mass (e.g., upper limb muscle) may be insufficient to significantly increase plasma IL-6 levels (Nosaka and Clarkson, 1996; Hirose et al., 2004; Bergfors et al., 2005; Pedersen and Febbraio, 2008). The sharpest increase in plasma IL-6 is typically observed in running, which involves several large muscle groups (Fischer, 2006; Pedersen and Fischer, 2007). On the other hand, although IL-8 mRNA increases up to 10-fold in response to exercise and up to twofolds with a pharmacological cocktail (palmitate, forskolin, and ionomycin) to mimicking exercise-stimulated contractions *in vitro* (Covington et al., 2016), circulating IL-8 increases only transiently after exhaustive exercise, suggesting that this myokine acts only locally in an autocrine/paracrine fashion (Nielsen and Pedersen, 2007). In this regard, neuromuscular stimulation of cultured human primary skeletal muscle cells (hSkMCs) increases IL-8 secretion by muscle cells (Scheler et al., 2013). The production of the myokine brain-derived neurotrophic factor (BDNF) is stimulated by some types of physical exercise. Acute aerobic exercise leads to increased BDNF plasma levels in an intensity-dependent manner, whereas acute strength exercise does not elicit this effect (Knaepen et al., 2010; Huang et al., 2014). Thus, rest periods between efforts, relative intensity and a limited amount of muscle mass mobilized and contracted simultaneously during strength exercise could limit the production of this and other myokines (Table 1).

**TABLE 1:** List of myokines potentially induced by muscle contraction and regular exercise (So et al., 2014; Schnyder and Handschin, 2015; Lightfoot and Cooper, 2016; Garneau and Aguer, 2019).

• Angiopoietin-like 4 (ANGPTL4) • Apelin • β-aminoisobutyric acid (BAIBA) • Brain-derived neurotrophic factor (BDNF) • Chemokine ligand and chemokine (C-X-C motif) ligand family • Decorin • Fibroblast growth factor 21 (FGF21) • Interleukin-6 (IL-6) • IL-8 • IL-10 • IL-13 • IL-15 • IL-18 • Irisin (FNDC5) • Musclin • Myonectin - C1q tumor necrosis factor a-related protein isoform 5 (C1QTNF5) • Myostatin • Leukemia inhibitory factor (LIF) • Secreted protein acidic rich in cysteine (SPARC) • Tumor necrosis factor-alpha (TNF-α)

Active skeletal fibers produce and release several myokines that act as hormones (Carson, 2017). These myokines released into the bloodstream exert well defined specific endocrine effects in different organs. This endocrine function of skeletal muscle may underlie numerous health benefits such as maintaining adequate body weight, reducing low-grade inflammation typical of chronic diseases, improving insulin sensitivity, protecting from tumor growth, and improving cognitive function. Therefore, physical exercise in which large muscle groups are mobilized at sufficient intensity and duration may produce benefits from a modulation of circulating myokines (He et al., 2018). Exercise also increases circulating small vesicles and exosomes as well as extracellular vesicles-packaged proteins involved in several biological functions (Whitham et al., 2018). Importantly, extracellular exosomes and extracellular matrix proteins might also be classified as myokines, particularly the extracellular matrix protein tenascin C, which is produced after electrical stimulation (Crameri et al., 2007) and affect muscle healing and regeneration (Fluck et al., 2008). However, the physical and metabolic limitations of some individuals will prevent them from undertaking physical exercise of sufficient intensity or duration to trigger such a myokine response.

Neuromuscular electrical stimulation (NMES) is based on applying in the transcutaneous electrical currents to a group of muscles, stimulating it to contract (Veldman et al., 2016). This method is usually employed as a passive “substitute” of dynamic training and acts as an “exercise emulator.” In fact, NMES can activate PGC-1α (the master regulator of mitochondrial biogenesis activated by endurance exercise) as well as the target of rapamycin (mTOR), which in turn activates insulin and IGF-1 receptors (Atherton et al., 2005). Accordingly, this strategy could be particularly useful in patients with paraplegia, tetraplegia, obesity and limited mobility, frail elderly, or any person needing prolonged bed rest.

This review describes the most relevant *in vivo* research findings linking NMES and endocrine myokine expression, revealing NMES as an ergo-mimetic agent, and discusses how this method of stimulating the production of plasma myokines can exert a beneficial effect on the pathophysiology of several conditions in patients with limited mobility. We have particularly focused on myokines for which sufficient scientific evidence is currently available.

## Myokine Production Throughout Neuromuscular Electrical Stimulation: a Review of *In Vivo* Evidence

Neuromuscular electrical stimulation may act as an efficient protector of muscle competence when subjects are unable or unwilling to engage in resistance or aerobic training programs. In accordance with this hypothesis, superimposed muscle contraction produced by electrical stimulators was found to enhance functional capacity in heart failure (HF) patients tested using the 6-min walk test (Nuhr et al., 2004; Karavidas et al., 2006). Similar results emerged from a meta-analysis of functional electrical stimulation in patients with chronic HF (Sbruzzi et al., 2010). Thus, NMES improved functional capacity (measured as VO_2__peak_) in HF patients to a similar extent as conventional aerobic training. The greatest improvements in the NMES group were detected in patients with a lower exercise capacity (Deley et al., 2008). A Cochrane systematic review (Jones et al., 2016) considered NMES a valid therapeutic intervention to improve muscle weakness in adults with conditions such as chronic obstructive pulmonary disease (COPD), chronic respiratory disease, chronic HF, or thoracic cancer. Likewise, NMES could be useful during recovery from injury (Caggiano et al., 1994; Wall et al., 2015), since it increases antioxidant capacity and decreases redox imbalance caused by disuse (Gondin et al., 2011a, b; Pellegrino et al., 2011).

## Interleukins

NMES behaves as a powerful stimulus to skeletal muscle with systemic consequences when undertaking considered at low to moderate workload. Compared to groups in which the only intervention was active cycling or passively applied NMES, cycling plus NMES produced the greatest increases in plasma IL-6 levels immediately and 30 and 60 min after the intervention, showing a significant interaction effect (intervention^∗^time) (partial η^2^ = 0.55; power = 0.99) (all *p* < 0.001) (Wahl et al., 2015).

The duration of exercise seems to be the most critical factor regulating the amplitude of the systemic IL-6 response. Since the intervention time was exactly the same for the three groups, these results may arise from the larger amount of muscle mass engaged in the intervention combining NMES and active cycling (Fischer, 2006).

Increased peripheral blood levels of IL-6 were measured after a single 30-min NMES session in healthy participants receiving bilateral lower extremity muscle stimulation in the quadriceps, tibialis anterior and gastrocnemius. This intervention produced a significant increase in peak IL-6 from the mean pre-NMES value [0.65 (0.89) to 1.04 (0.89) pg ml^–1^, *P* = 0.001], and a significant decrease in interleukin-10 (IL-10) [0.08 (0.07) to 0.02 (0.02) pg ml^–1^, *P* = 0.041] and TNF-α [2.42 (0.54) to 2.16 (0.59) pg ml^–1^, *P* = 0021]. Significantly higher mean values of IL-6 were also observed after NMES throughout the full 120-min period (Truong et al., 2017).

Although the magnitude of change was not impressive, these results are in line with similar responses to exercise observed in healthy adults (Greiwe et al., 2001; Steensberg et al., 2002). Truong et al. (2017) demonstrated a clear relationship between exercise-induced release of IL-6 and TNF-α, thus supporting its putative anti-inflammatory role. Specifically, IL-6 seems to exert its anti-inflammatory actions during low to moderate exercise, through induction of IL-1ra transcription, which in turn inhibits the pro-inflammatory cytokine IL-1. Moreover, IL-6 increases the production of IL-10, which inhibits lipoprotein saccharide-stimulated production of the pro-inflammatory cytokines TNF-α, IL-1α, and IL-1β (Steensberg et al., 2003; Fischer, 2006).

Chronic conditions such as COPD may lead to a pro-inflammatory state. In these situations, IL-6 may be increased in the presence of increased TNF-α expression. Akar et al. (2017) studied COPD exacerbation during intubation in hospital, and found significantly reduced plasma IL-6 levels after a rehabilitation program based on NMES and active mobilization [5.70 (1.70–13.70) – 1.20 (0.50–2.70) (*P* = 0.015)], and on NMES alone [3.35 (0.70–14.18) – 1.20 (0.50–6.70) (*P* = 0.068)]. The intervention group only undergoing active mobilization showed increased IL-6 levels without significant differences (Akar et al., 2017). Similarly decreased levels of IL-8 were found in a NMES and active mobilization group [13.64 (1.47–23.70) – 2.35 (0.80–17.63) (*P* = 0.017)], and a NMES alone group [6.13 (2.35–25.00) – 3.92 (0.80–17.63) (*P* = 0.017)]. This time, the active mobilization intervention group showed no significant differences in IL-8. Although no significant difference was reported, NMES and active mobilization, as well as NMES alone, were effective in increasing IL-10, whereas the active mobilization alone group showed reduced IL-10 values. This downregulation of IL-6 and IL-8, and upregulation of IL-10, could be consequent to a reduced proinflammatory state mediated by muscle contraction.

## Brain-Derived Neurotrophic Factor

Miyamoto et al. (2018) carried out several studies in healthy subjects and those with type 2 diabetes mellitus. These authors reported significantly improved plasma BDNF concentrations after an 8-week period of NMES training and after a single bout of NMES, respectively. In this study, a single 30 min bout of NMES significantly increased plasma BDNF levels (pre-NMES: 150.5 ± 126.7 vs. post-NMES: 250.5 ± 131.1 pg mL^–1^; *p* = 0.017); this effect was similar to that observed in subjects completing a 30-min cycling ergometer exercise test at 60% VO_2__peak_ (post-NMES: 250.5 ± 131.1 vs. post-exercise: 268.6 ± 123.8 pg ⋅ mL^–1^; *p* = 0.908). However, this acute response was not associated with an improvement in cognitive function (Miyamoto et al., 2018). The 8-week protocol of NMES training in subjects with type 2 diabetes was able to induce a significant increase in plasma BDNF (pre-NMES: 117.0 ± 40.4 vs. post-NMES 245.5 ± 51.2 pg/ml; *p* = 0.026) compared to participants allocated to the control group and who showed a decline in plasma BDNF during the 8-week period without NMES. The NMES intervention also induced a greater reduction in the body fat percentage and fasting glucose concentrations than in the control group (Miyamoto et al., 2018).

Kimura et al. (2019) compared voluntary exercise and NMES-induced muscle contraction with the same integrated force measured using electro-myographic technology, and observed that the increase in serum BDNF in the NMES group was higher than that in the voluntary exercise group (18625.6 ± 4173.5 pg/ml, *p* = 0.003 vs. 15103.0 ± 4177.9 pg/ml, *p* = 0.004). NMES could therefore be even more effective than active exercise using the same integrated force to increase serum BDNF.

Several animal experiments have also examined the relationship between BDNF and exercise. In 2017, Dalise et al. (2017) obtained surprising results in Sprague-Dawley rats. These authors compared serum myokines such as vascular endothelial growth factor-A (VEGF-A), insulin-like growth factor-1 (IGF-1), Klotho (i.e., an anti-aging single-pass membrane protein), and BDNF produced in response to different intensities (low, medium, or high) of active exercise and analogous NMES interventions. NMES did not modify IGF-1 levels yet led to a modest increase in plasma Klotho concentrations in the low- and high intensity interventions. Notably, after a medium-intensity session of NMES, serum BDNF underwent a dramatic eightfold increase (*p* = 0.01).

Maekawa et al. (2018) found that 50 repeated maximal electrically evoked-isometric contractions in unconscious rats were effective at increasing BDNF protein expression and activate its hippocampus receptor. These findings could provide reliable evidence of an alternative means of communication between muscle and different organs, additional to endocrine interactions.

## Myostatin

Myostatin is an inverse modulator of muscle mass in animals and humans (McPherron and Lee, 1997; Schuelke et al., 2004; Mosher et al., 2007), inhibiting mTOR signaling (Rodriguez et al., 2011). Wall et al. (2012) showed that the expression of myostatin mRNA declined significantly after 60 min of NMES in the lower limbs, coinciding with a significant increase in MyoD mRNA expression. These results are consistent with an anabolic stimulus following a bout of resistance exercise. Dirks et al. (2014) reported that the expression of myostatin mRNA was lower after NMES compared to baseline or a control group not receiving electrical stimulation. The expression of MyoD mRNA was also increased after NMES compared to values recorded at baseline and in the non-NMES group.

## Gh and Igf-1 Signaling

In an experimental study in rats subjected to sciatic neurectomy to reproduce adverse effects such as disuse amyotrophy and cortical bone loss (Feng et al., 2016), 30-min NMES sessions 5 days per week for 9 weeks downregulated mRNA expression levels of myostatin and upregulated those of mechano-growth factor (MGF) and insulin-like growth factor 1 (IGF-1).

In a treadmill study in Sprague-Dawley rats, resistance training (based on superimposed NMES-induced isometric contraction) led to increased and maintained IGF-1 and GLUT-4 translocation compared to aerobic exercise, suggesting a potential role of NMES resistance training as a regulator of glucose metabolism (Kido et al., 2016).

The growth hormone (GH) response to exercise is a driving force of anabolic protein synthesis linked to strength and muscle mass enhancement. NMES is a valid and efficient tool to stimulate a hormone response in healthy subjects (Aldayel et al., 2010; Wahl et al., 2014). Collectively, evidence so far suggests that NMES mediates a protective effect on muscular structural integrity and functional capacity via autocrine and paracrine mechanisms.

## Dose-Effect Relationship Between Contraction Intensity and Myokines

Unfortunately, no information exists on a minimal contraction and/or number of sessions needed to stimulate myokines’ secretion. However, although at present there are no precise data about the dose-response relationship between NMES-induced contraction intensity and myokines secretion, higher intensities of muscle contraction provoked by NMES improved muscle function in patients after an anterior cruciate ligament reconstruction (Snyder-Mackler et al., 1994). There is also a linear dose-response relationship between the increase in energy expenditure and the intensity of NMES in healthy subjects (Hsu et al., 2011) as well as with muscle function in patients with rheumatoid arthritis (Almeida et al., 2018). Likewise, a linear dose-response relationship was observed between NMES intensity and quadriceps strength and voluntary activation in subjects who received NMES after total knee arthroplasty, although there was no evidence of an association with muscle cross-sectional area (Marmon and Snyder-Mackler, 2011). Therefore, it is likely that there is a dose-effect relationship between NMES intensity and other variables, but additional investigations are still needed to elucidate the dose-effect relationship between NMES intensity-frequency-duration-muscle/s and myokines’ secretion.

## Potential Drawbacks and Limitations of Using Nmes

NMES-provoked muscle damage characterized by histological alterations in muscular and connective tissue, creatine kinase (CK) activity increases, declines in muscle strength, and delayed onset muscle soreness has been recently reported (Nosaka et al., 2011). Several cases of rhabdomyolysis induced by NMES have been reported (Guarascio et al., 2004; Kastner et al., 2015; Johannsen and Krogh, 2019). NMES might increase myostatin (also named GDF-8; growth/differentiation factor 8), an inhibitor of skeletal muscle growth, and GDF-15 (growth differentiation factor 15), suggesting thus that excessive NMES could damage muscles (Bloch et al., 2014). Nevertheless, it seems that pre-conditioning muscles by isometric contractions or submaximal eccentric might attenuate NMES-provoked muscle damage (Nosaka et al., 2011).

On the other hand, a rehabilitation program including NMES must be accompanied by functional task to guarantee the eventual success of the intervention (Azman and Azman, 2017). Although there is controversy regarding the additional benefits of “superimposed” NMES in trained subjects vs. voluntary exercise alone, the former could be effective in untrained subjects or in patients who cannot mobilize appropriately (Paillard et al., 2005). Superimposed NMES seems more effective than voluntary exercise alone for the prevention of muscle atrophy, maintenance of muscle oxidative capacity and prevention of strength loss, and to recover knee function and gait kinematics after ligament surgery (Eriksson and Haggmark, 1979; Snyder-Mackler et al., 1991). A large muscle mass and a higher strain to skeletal muscles than normal are needed to increase the secretion of certain myokines (Wahl et al., 2015): this is the reason why superimposed NMES might be more effective to induce a higher local muscle stimulus for myokine secretion. Finally, voluntary exercise is at least as beneficial as superimposed NMES, although the latter could produce additional benefits in weaker muscles (Hartsell, 1986).

## Most Commonly Neuromuscular Electrical Stimulation Protocols Used in Humans to Evaluate Circulating Myokine’s Secretion

Table 2 summarizes the most commonly NMES protocols used in human investigations. In general, NMES sessions last ∼20 to 60 min, with a stimulation frequency between 4 and 2000 Hz, pulse (biphasic rectangular pulses) duration of 50–1000 μs, with the highest tolerable intensity to maximize force production, performed every day in patients with activation neural deficits, and alternate days to produce hypertrophy in the affected muscle when the neural deficits have improved (Maffiuletti et al., 2018).

**TABLE 2:** Summary of the different NMES protocols most commonly used to evaluate circulating myokine’s secretion in humans.

**Study**	**Type**	**Frequency**	**Pulse width**	**Intensity**	**Time**	**Total number of sessions**	**Stimulated zone**	**Others**
Dirks et al., 2014	Biphasic symmetrical rectangular-wave pulses	Warm-up: 5Hz Stimulation period: 100Hz Cooling down phase: 5Hz	Warm-up: 250 μs Stimulation period: 400 μs Cooling down phase: 250 μs	Subjects set the intensity of the stimulation to a level at which full contractions of m quadriceps femoris were visible and palpable	Warm-up: 5′ Stimulation period: 30’ Cooling down phase: 5′	10 sessions (2 sessions per day/5-day period)	Self-adhesive electrodes placed on the distal part at the m. rectus femoris and the m. vastus lateralis	Volunteers were subjected to 5 days of one-legged knee immobilization
Wahl et al., 2015	Bipolar rectangular pulse continuously applied	60 Hz	400 μs	Progressively increased at maximum tolerated	60′	One unique session	Circular electrodes around the thigh and the calf and laminar electrodes in the gluteal zone	The electrical stimulation was continuous and independent from the cycling
Akar et al., 2017	Symmetrical biphasic squared waveform, pulsed (ramp- up 1.5 s, 6 s duration contraction and 0.75 ramp-down; the rest time was not reported and the duty-cycle rest was unknown)	50 Hz	Not reported	Until visible contraction was obtained, 20-25 mA (depending on patient tolerance).	Not reported	20 sessions (5 days per week, 4 weeks).	bilateral upper extremity (deltoid) and bilateral lower extremity (quadriceps).	Volitional contraction was not allowed for the patients
Dalise et al., 2017	Interference wave at an amplitude-modulated frequency of 20 Hz, on-off ratio 4.5:4.5 s (ramp-up 1 s and ramp-down 0.5 s)	2000 Hz	50 μs	Progressively increase to the highest tolerated intensity during the experiment	20′	One unique session	Bilateral lower limbs (quadriceps) were stimulated alternately	–
Truong et al., 2017	Pulsed asymmetrical biphasic waveform (2 s ramp-up, 5 s duration contraction and <1 s ramp-down, 8/18 duty-cycle)	50 Hz	400 μs for quadriceps and 250 μs for tiabialis anterior and gastrocnemius	Until visible contractions were obtained	30′	One unique session	Quadriceps, tibialis anterior and gastrocnemius	Electrical stimulation of the tibialis anterior and gastrocnemius alternated to stimulate physiologic volitional contraction in order to prevent discomfort
Maekawa et al., 2018	Pulsed rectangular waveform (3 s stimulation time and 7 s rest period Duty-cycle: 3/10)	100 Hz	1000 μs	3 to 5 V	10 contractions per set, 5 sets with an interval of 3’	One unique session	Triceps surae muscle.	–
Miyamoto et al., 2018	Pulsed rectangular monophasic wave form	4 Hz	250 μs	Progressively increased until maximum tolerated	30’ (single bout of NMES) and 40’ for the 8 weeks protocol	One unique session and 5 days per week, for 8 weeks (40 sessions), respectively	Circular electrodes were used around the thigh and the calf and laminar electrodes were placed in the gluteal zone	–
Kimura et al., 2019	Interference wave at an amplitude-modulated frequency of 20 Hz, on-off ratio 4.5:4.5 s (ramp-up 1 s and ramp-down 0.5 s)	2000 Hz	0.05 ms	Progressively increase to the highest tolerated intensity during the experiment	20’	One unique session	Bilateral lower limbs (quadriceps) were stimulated alternately	–

## Conclusion

**FIGURE 1:**
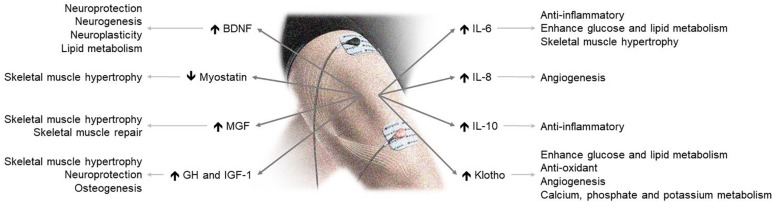
NMES-induced myokine production and its effects on health. BDNF, brain-derived neurotrophic factor; GH, growth hormone; IGF-1, insulin-like growth factor 1; MGF, mechano-growth factor.

The available data show that NMES is safe and efficient to improve muscle strength, functional capacity and quality of life. Evidence mounts that NMES stimulates the secretion of circulating myokines with clinically relevant endocrine, paracrine and autocrine consequences (Figure 1). Accordingly, NMES may have many potential effects and applications in health and disease. Although further data are needed, including measurements of other central myokines (Table 1), current evidence suggests that NMES may be a useful physical exercise substitute for myokine modulation eliciting skeletal-muscle hypertrophy and neuromuscular adaptations.

## Limitations

This review was not planned to be a comprehensive, systematic, and/or cumulative review of existing evidence regarding NMES pros and cons, but a narrative review aiming to give a broad overview on NMES clinical use and address whether there is enough scientifically valid evidence to recommend NMES as an effective physical exercise substitute or complement to induce myokine production, and delineate the potential applications of NMES in health and disease. Therefore, there was not a predefined protocol-based search method of the bibliography which might involve subjective selection bias. Unfortunately, there are not many scientific studies that specifically evaluate the effects of NMES on circulating myokine levels. For that reason, we have limited this review to certain myokines for which sufficient scientific evidence is currently available. Therefore, additional studies would be needed for particularly assessing the potential role of NMES in regulating the expression of a wider variety of myokines.

## Author Contributions

FS-G conceived and coordinated the writing of the manuscript. FS-G, SL-L, CR-M, NM, GL, and HP-G contributed to the writing of the manuscript. HP-G prepared the figure.

## Conflict of Interest

The authors declare that the research was conducted in the absence of any commercial or financial relationships that could be construed as a potential conflict of interest.
